# Effect of Time Interval and Frequency of Hospitalization Because of Fluid Overload on Survival in Peritoneal Dialysis: Thailand Experience

**DOI:** 10.34067/KID.0000000576

**Published:** 2024-09-11

**Authors:** Jaruwan Thuanman, Pornpen Sangthawan, Kavin Thinkhamrop, Bandit Thinkhamrop, Jadsada Thinkhamrop, Siribha Changsirikulchai

**Affiliations:** 1 Epidemiology and Biostatistics Program, Faculty of Public Health, Khon Kaen University, Khon Kaen, Thailand; 2 Data Management and Statistical Analysis Center (DAMASAC), Faculty of Public Health, Khon Kaen University, Khon Kaen, Thailand; 3 Division of Nephrology, Department of Medicine, Faculty of Medicine, Prince of Songkla University, Hat Yai, Thailand; 4 Health and Epidemiology Geoinformatics Research (HEGER), Faculty of Public Health, Khon Kaen University, Khon Kaen, Thailand; 5 Department of Obstetrics and Gynecology, Faculty of Medicine, Khon Kaen University, Khon Kaen, Thailand; 6 Division of Nephrology, Department of Medicine, Faculty of Medicine, Srinakharinwirot University, Nakhonnayok, Thailand

**Keywords:** chronic dialysis, CKD, chronic kidney failure, clinical epidemiology, dialysis, peritoneal dialysis, RRT

## Abstract

**Key Points:**

- High mortality was found in patients on peritoneal dialysis who were hospitalized early or frequently because of fluid overload.
- Interval time and frequency of hospitalization because of fluid overload are clinical indicators for the need of intensive fluid management.

**Background:**

Fluid overload (FO) is common and linked to high mortality in patients undergoing peritoneal dialysis (PD). This study evaluates the effect of time interval and frequency of FO-related hospitalizations on mortality and patient survival rates in patients on PD.

**Methods:**

Data from patients on PD voluntarily registered in the Database of Peritoneal Dialysis in EXcel were reviewed. We included patients who started PD between January 2008 and December 2018, had a history of FO-related hospitalizations after starting PD, and were followed until December 2020 or death. We analyzed the time interval to the first FO-related hospitalization after starting PD, number of such hospitalizations, and cumulative FO-free time. Mortality and patient survival rates were calculated, and multiple Cox regression identified factors associated with mortality.

**Results:**

Among 1858 patients hospitalized because of FO, those hospitalized within 12 months of starting PD or with <12 months of cumulative FO-free time had high mortality rates of 38.8 and 40.3 per 100 patient-years, respectively. One-year survival rates were 70.1% for those with a time to first FO-related hospitalization within 12 months of starting PD and 68.7% for those with <12 months of cumulative FO-free time. Adjusted hazard ratios were 2.92 (2.31–3.69) for a cumulative FO-free time of <12 months, 1.53 (1.18–1.99) for time to first FO-related hospitalization within 12 months and 1.05 (1.03–1.07) per FO-related hospitalization.

**Conclusions:**

The time interval to the development of FO significantly affects mortality in patients undergoing PD.

**Podcast:**

This article contains a podcast at https://dts.podtrac.com/redirect.mp3/www.asn-online.org/media/podcast/K360/2024_11_21_KID0000000576.mp3

## Visual Abstract

**Figure:**
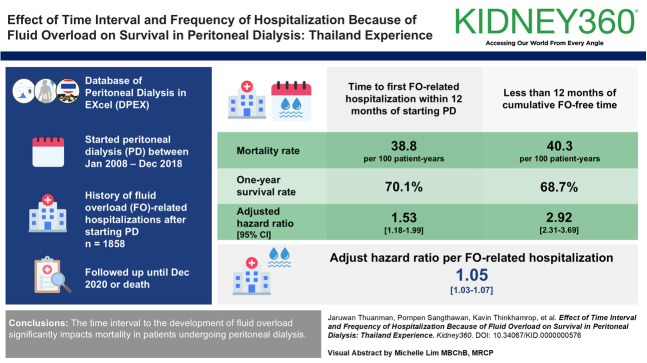

## Introduction

Fluid overload (FO) is an important cause of mortality and transitioning to hemodialysis from peritoneal dialysis (PD).^1–3^ It is commonly found in patients on PD, as shown in previous studies.^4^ Data from the Initiative for Patients Outcomes in Dialysis-PD study demonstrated that FO presented before the initiation of dialysis, but tended to improve from baseline over the next 6 months and then stabilize over time.^5^ However, a recent single-center study showed that FO at the initiation of dialysis persisted over time in a substantial proportion of patients on PD.^6^ Previous studies identified risk factors of FO in patients on PD as increasing age, diabetes, male sex, hypoalbuminemia, protein energy wasting, and inadequate dialysis.^5–7^ These studies used bioimpedance analysis (BIA) devices to identify FO. The BIA device could assess FO in asymptomatic patients and guide management to improve FO.^6–8^ It has been shown that the time average of FO defined by relative hydration index using the BIA device was associated with increased mortality.^6^ However, the routine use of the BIA device to guide management of FO did not show improvement in 1-year patient survival and technique survival nor provide additional benefits in controlling fluid, preserving residual kidney function (RKF), and preventing cardiovascular events in patients on PD.^9,10^ In addition, the BIA device is available in some dialysis centers with limitations on repeat measurements. It is necessary to find other clues to guide management of FO, broaden its availability, and provide prognosis in clinical practice. In this study, we evaluate the effect of time interval and frequency of FO on mortality and patient survival rates in patients on PD. We selected patients on PD who were hospitalized because of FO because they had moderate or severe FO.

## Methods

### Study Population

**Figure 1:**
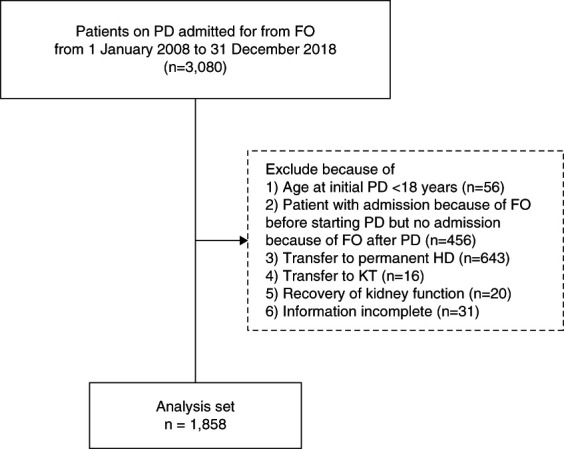
**Flow of the study population.** FO, fluid overload; KT, kidney transplantation; PD, peritoneal dialysis; HD, hemodialysis.

We performed a retrospective cohort observational study by reviewing data of patients on PD who were registered in the Database of Peritoneal Dialysis in EXcel (DPEX). The DPEX registry is designed to help PD units monitor outcomes and improve care quality through continuous quality improvement. PD units voluntarily register to use DPEX without any associated costs, and the data are entered naturally without selection bias. There were 92 PD units with 21,712 patients registered in the DPEX program during the studying period. The distribution of PD centers and number of patients classified by region are provided in Supplemental Table 1. We included patients who were at least 18 years of age, started PD between January 2008 and December 2018, and had a history of hospitalizations because of FO after starting PD. Patients who were admitted because of FO before starting PD but without a history of admission from FO after starting PD, transferred to permanent hemodialysis, underwent kidney transplantation, had recovery of kidney function, or had incomplete information were excluded from the study. A total of 1858 patients met the eligibility criteria. Figure 1 demonstrates the flow of the study population. This study was approved by the Institutional Review Committee for Research in Human Subjects at Khon Kaen University and Srinakharinwirot University (SWUEC-481/2563X).

### Data Collection and Variables

Baseline characteristics of patients were recorded, including age at the start of PD, sex, educational levels, type of reimbursement from health payment, causes of ESKD, comorbidities, serum creatinine, and eGFR at the time of initiating PD, time on PD therapy, and history of admission from FO within 12 months before starting PD. CKD Epidemiology Collaboration creatinine equation was applied to calculate the eGFR.^11^ Comorbidities were classified as diabetes, cerebrovascular disease, cardiovascular disease, liver disease, gastrointestinal disease, cancer, and airway disease according to the International Statistical Classification of Diseases and Related Health Problems, Tenth Revision codes. The frequency of admission because of FO after starting PD, length of stay per admission, cost of treatment per admission, time interval from starting PD to first admission because of FO, and cumulative FO-free time interval were calculated.

### Definition and Outcome

Patients were followed until the date of censoring at the end of December 2020 or the date of death while on PD. The primary outcomes were patient survival and all-cause mortality rates. Admission because of FO was identified by using various combinations of International Statistical Classification of Diseases and Related Health Problems, Tenth Revision codes for FO, pulmonary edema, and pleural effusion diagnosed at discharge. The time interval from starting PD to first admission because of FO was calculated from the date of starting PD to the date of first admission because of FO and classified every 12 months. The cumulative FO-free time interval was analyzed for patients with at least two episodes of admission. It was calculated by summing the number of days the patient remained free from admission because of FO. This calculation excluded the days after the last admission for FO until the date of death or the end of the study (Supplemental Figure 1). The intervals were classified in 12-month increments.

### Statistical Analyses

The analysis was performed with STATA version 15 (Stata Corp, College Station, TX). The categorical variables were presented in numbers and percentages. The continuous variables were demonstrated as median with interquartile range (IQR). 95% confidence interval (CI) was analyzed on the basis of normal approximation to binomial distribution. The mortality rates per 100 person-years since initiating PD, and its 95% CI were calculated on the basis of Poisson distribution assumption. Patient survival was analyzed by using the Kaplan–Meier method. The risk factors of all-cause mortality were investigated by a multivariate Cox proportional hazard model, including all the significant variables in a backward manner from the univariate analysis and/or those variables considered clinically relevant according to the current literature. A *P* value of <0.05 was considered statistically significant.

## Results

### Patient Characteristics

Table 1 presents the characteristics of 1858 patients on PD who had a history of hospitalization because of FO after the initiation of PD. The number of male and female patients was not significantly different. Most of the patients were illiterate or had only a primary school education. The main type of health care reimbursement was universal coverage. Diabetic nephropathy was the cause of ESKD in 53.5% of the patients. In addition, 46.9% of patients had comorbid cardiovascular or cerebrovascular diseases. The median (IQR) eGFR at the time of starting PD was 4.6 (3.3–6.7) ml/min per 1.73 m^2^. The median (IQR) duration on PD therapy was 29.5 (15.7–48.1) months. The rate of peritonitis (PN) was <0.4 episodes per year at risk in the study cohort (Supplemental Table 2), which is lower than the PN rate recommended by the International Society for Peritoneal Dialysis PN guideline recommendations: 2022.^12^

**Table 1:** Characteristics of patients on PD admitted because of fluid overload

Characteristics	All Patients (*N*=1858 Patients)
Age at PD initiation (yr) median (IQR)	55.7 (46.7–64.1)
Sex, male: female, no. (%)	920 (49.5): 938 (50.5)
**Education level, no. (%)**	
Illiterate and primary school	1512 (81.4)
Secondary school or higher	346 (18.6)
**Type of reimbursement, no. (%)**	
Universal coverage	1716 (92.4)
Others	142 (7.6)
**Cause of ESKD, no. (%)**	
Diabetic nephropathy	994 (53.5)
Analgesic nephropathy	168 (9.0)
Hypertension	477 (25.7)
Obstructive nephropathy	20 (1.1)
Unknown	199 (10.7)
**Comorbidity, no. (%)**	
Cerebrovascular disease	431 (23.2)
Cardiovascular disease	440 (23.7)
Liver disease	77 (4.1)
Gastrointestinal disease	74 (4.0)
Airway disease	105 (5.7)
Creatinine at start PD (mg/dl), median (IQR)	9.9 (7–13.4)
eGFR at start PD (ml/min per 1.73 m^2^) median (IQR)	4.6 (3.3–6.7)
Time on PD therapy (mo) median (IQR)	29.5 (15.7–48.1)
Patients with a history of admission from FO within 12 mo before starting PD, no. (%)	627 (33.7)
**Frequency of admission from FO after starting PD, no. (%)**	
One admission	823 (44.3)
Two admissions	493 (26.5)
Three admissions	193 (10.4)
More than three admissions	349 (18.8)
Median (IQR); (min: max)	2 (1–3); (1:23)
Length of stay per admission (d) median (IQR)	5 (3–8)
Cost per admission (baht) median (min:max)	8350.00 (11,953.90: 230,706.30)
**Time interval from starting PD to first admission due to FO, mo, no. (%)**	
0–12	1211 (65.2)
12–24	256 (13.8)
24–36	170 (9.2)
More than 36	221 (11.9)
**Cumulative FO-free time interval, mo, no. (%)**	
0–12	460 (44.4)
12–24	217 (21.0)
24–36	134 (12.9)
More than 36	224 (21.7)

FO, fluid overload; IQR, interquartile range; PD, peritoneal dialysis.

A total of 33.7% of patients had a history of admission because of FO within 12 months before starting PD. The number of patients hospitalized because of FO at least twice after starting PD was 1035 (55.7%). The median (IQR) number of admissions because of FO after starting PD was two (1–3). One patient was hospitalized 23 times for FO. The median (IQR) length of stay per admission was 5 (3–8) days. The median cost of treatment paid by the National Health Security Office (NHSO), calculated according to the adjusted relative weight (adjusted RW) on the basis of disease-related group, was 8350.00 baht (238.6 USD; 1 USD=35 baht). The number of patients first hospitalized because of FO within 12 months after starting PD was 1211 (65.2%). The number of patients who had a cumulative FO-free time interval of 12 months or less, 12–24, 24–36 months, and more than 36 months was 460 (44.4%), 217 (21.0%), 134 (12.9%), and 224 (21.7%), respectively.

### Patient Survival and Mortality Rate

**Figure 2:**
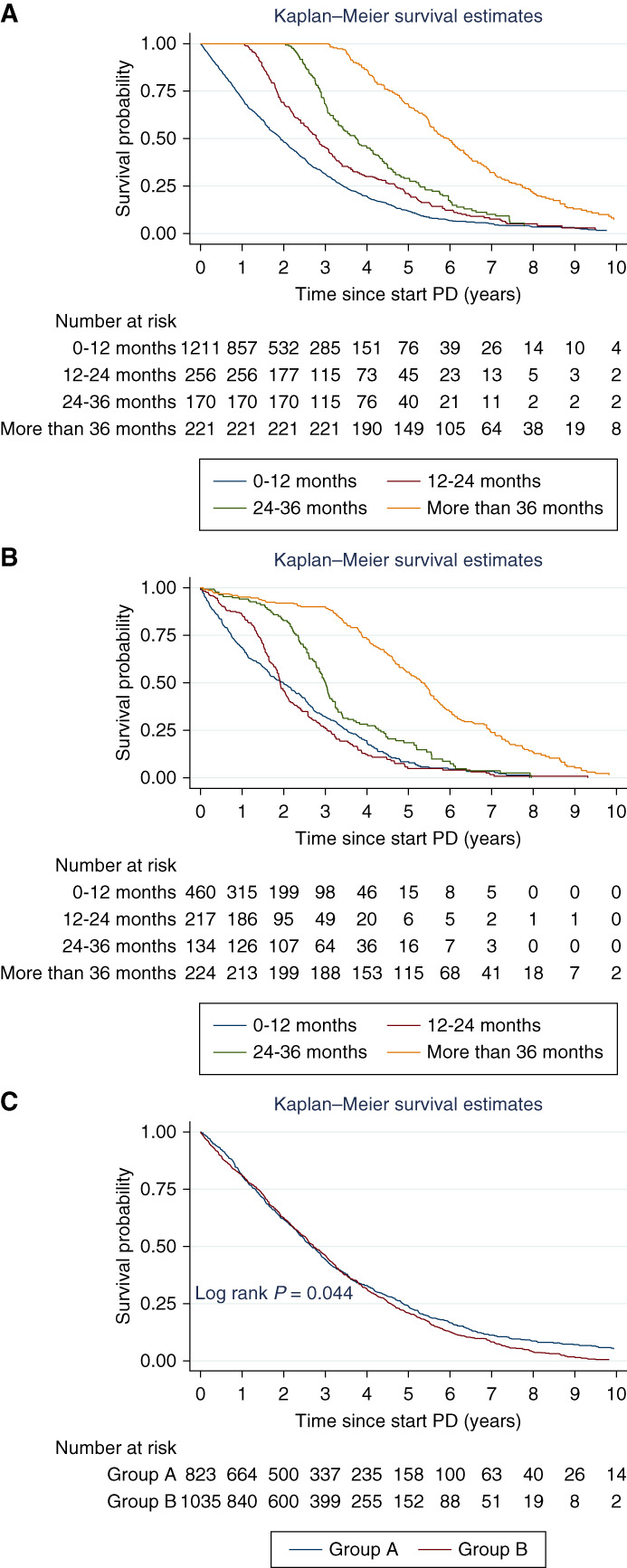
**Patient survival rates according to time interval and frequency of FO in patients undergoing PD.** (A) Time interval to first admission for FO. (B) Cumulative FO-free time interval. (C) Frequency of admission because of FO classified into two groups. Group A: one admission because of FO. Group B: admission because of FO at least two episodes.

Figure 2 shows patient survival rates according to time intervals and frequency of FO in patients undergoing PD. The 1-, 3-, and 5-year survival rates for patients who had their first admission time interval due to FO within 12 months after starting PD were 70.1% (95% CI, 68.3% to 73.4%), 31.3% (95% CI, 28.6% to 34.1%), and 11.7% (95% CI, 9.7% to 14.1%), respectively (Figure 2A). Patients with a history of early admission tended to have a higher percentage of ESKD due to diabetes, along with increased comorbidities in cardiovascular, cerebrovascular, and gastrointestinal diseases, including a higher total number of comorbidities (Supplemental Table 3). RKF could not be evaluated because of the large amount of missing data.

Patient survival rates were lower for those with a cumulative FO-free time interval of 12 months or less and 12–24 months compared with those with longer free times (Figure 2B). The 1-, 3-, and 5-year survival rates for patients with a cumulative FO-free time interval of 12 months or less were 68.7% (95% CI, 64.2% to 72.7%), 31.9% (95% CI, 27.3% to 36.4%), and 8.4% (95% CI, 5.3% to 12.4%), respectively.

**Figure 3:**
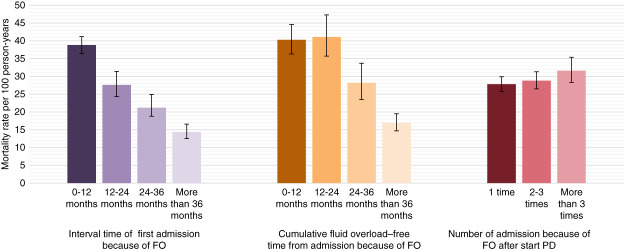
**Mortality rates according to time interval and frequency of admission because of FO in patients undergoing PD**.

For patients hospitalized because of FO at least twice, survival rates were similar to those hospitalized once, with differences in survival rates emerging after 5 years (Figure 2C). Figure 3 illustrates the mortality rates of patients on PD according to the time interval and frequency of admission because of FO. The mortality rate was 38.8 per 100 person-years for patients whose first admission because FO occurred within 12 months, compared with 27.6, 21.2, and 14.4 per 100 person-years for those with intervals of 12–24 months, 24–36 months, and more than 36 months, respectively. A high mortality rate was observed in patients with a cumulative FO-free time interval of 12 months or less, and those with free times of 12–24 months, followed by those with a free time of 24–36 and more than 36 months, with rates of 40.3, 41.1, 28.2, and 16.9 per 100 person-years, respectively. The mortality rate of patients with one admission from FO was 27.8 per 100 person-years and with 2–3 episodes was 28.8 per 100 person-years. It was 32.6 per 100 person-years in those with admission more than three episodes.

### Factors Associated with All-Cause Mortality

**Figure 4:**
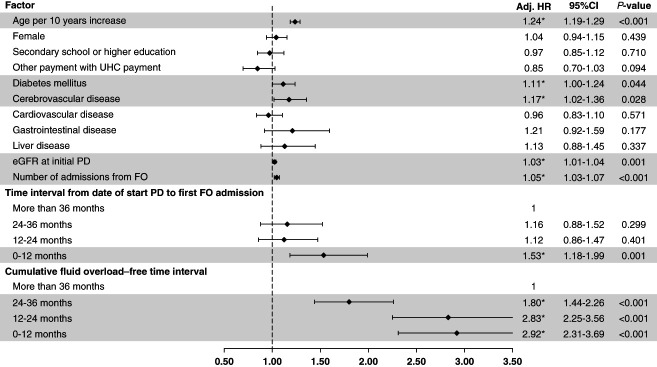
**Factors associated with all-cause mortality in PD patients with a history of admission because of FO.** CI, confidence interval; Adj.HR; adjusted hazard ratio; UHC, universal health coverage.

Figure 4 shows the adjusted hazard ratio for factors associated with all-cause mortality. A cumulative FO-free interval of 12 months or less was the strongest factor associated with death, followed by free intervals of 12–24 and 24–36 months. Other factors associated with mortality in patients with FO included time interval to the first admission because of FO within 12 months, increasing age, ESKD caused by diabetes, comorbidity in cerebrovascular disease, eGFR at the time of PD initiation, and the number of admissions because of FO.

## Discussion

This study illustrates the effect of specific time intervals and number of hospitalizations because of FO on mortality and patient survival in those undergoing PD. It emphasizes the importance of managing FO effectively, especially in patients at high risk of developing FO.

FO leads to hypertension and left ventricular hypertrophy, both established risk factors of cardiovascular mortality.^13–15^ Previous studies have demonstrated that strict fluid control can improve cardiac function, arterial stiffness, and BP in patients on dialysis.^16–18^ A substantial proportion of patients in our study had a history of FO before starting PD, corresponding to previous studies showing that moderate-to-severe FO occurred in most patients before starting PD.^5^ Time interval to the first admission because of FO occurred within 12 months, frequent admissions, and a short cumulative FO-free interval were key indicators in clinical practice that these patients had persistent severe FO and an increased risk of mortality, similar to those identified with FO by a BIA device.^6^ This implies that the management of FO, especially in these patients, should be aggressive and effective.

Various strategies are used to manage FO which include dietary salt restriction, maintaining RKF, using appropriate diuretics in patients with RKF, increasing ultrafiltration by using icodextrin, adding a manual daytime exchange in patients undergoing night-time automated PD, or switching to hemodialysis.^19–21^ A previous study showed that diabetic patients had more FO, higher dialysis glucose load and dialysis dose, and more total fluid removal than nondiabetic patients, which improved after restricting salt and water intake.^22^ Another study implemented a self-management strategy to improve FO, which included intensively educating patients to restrict salt and fluid intake, improving patient's ability to monitor their fluid status, improving ability to cope with a sudden FO, and closely following up to motivate patients.^23^

Achieving fluid balance is a challenging issue, especially in PD patients with diabetes and vulnerable social characteristics. Our patients have low education levels, the majority are covered by universal health care, a significant portion suffer from diabetic nephropathy, and many have comorbid cardiovascular or cerebrovascular diseases. In addition, these factors are related to cognitive impairment, which affects to low compliance levels in terms of low adherence to dietary salt restriction, medications, and dialysis regimens.^24^ Therefore, they require personalized interventions suitable to their comprehensive conditions. Health literacy programs to raise awareness about choosing suitable foods for dietary salt restriction, self-monitoring of FO, and coping skills to manage FO or fluid depletion should be designed and tailored to match the patients' education levels. Patients with cognitive impairment or low compliance to treatment should be identified early and managed properly.

The use of diuretics increases urine volume and sodium excretion.^25^ However, large doses are required, which carry a risk of adverse effects and are ineffective in patients who have lost RKF, defined by urine output <100 ml/d. Several factors have been reported to affect the decline of RKF, including diabetes, chronic inflammation, low serum albumin, and proteinuria.^26–29^ The use of diuretics to relieve symptoms of FO in our patients has limitations. It may improve FO in the early and short periods on PD because most had diabetes as the cause of ESKD and high comorbidity, which means that they had chronic inflammation.

Selecting the appropriate type of PD fluid and prescription is associated with improving fluid status. Icodextrin PD solution has been reported to increase sodium removal and ultrafiltration.^30–32^ Longer nocturnal dwell times and performing a supplementary diurnal exchange were associated with higher sodium removal rates in patients on automated PD.^33^ Characteristics of the peritoneal membrane obtained from the peritoneal equilibration test (PET) guide individualized PD prescription.^34^ In our study, PET data were available for 402 of 1858 patients. Therefore, performing PET should be emphasized for these patients to provide high-quality care. Patients in this study who had incident ESKD started with PD under the PD First policy, and none of them transitioned from hemodialysis to PD. At present, the policy for dialysis in patients using universal health coverage has shifted from PD First to shared decision making, resulting in most patients starting with hemodialysis. We predict that they may shift to PD after failing hemodialysis, likely due to early unavailable vascular access or comorbidities that are not suitable for hemodialysis. This means that PD would be the last dialysis modality to save their lives. Icodextrin should be considered under the universal health coverage benefit, at least for patients at high risk of developing severe and persistent FO by using clinical indicators, as shown in this study.

We provided the information on the cost per admission of early FO, but it did not represent the actual expenses incurred during hospitalization. The cost of treatment was paid by the NHSO according to the adjusted RW, which may depend on the budget left at the end of each fiscal year. Hospitals had to absorb the extra cost that was not paid, which might negatively affect the care process.

Our study had some limitations. First, some data affecting FO, such as residual urine volume, rate of decline in RKF, number of hypertonic uses, and rate of noncompliance, were not available. We could not analyze the effect of glucose load on FO or the rate of decline in residual urine volume. A home visit program could help to identify compliance of patients to the treatment, especially in the setting of home-based therapy. Second, most of the patients did not have PET information, so we could not identify the relation between FO and peritoneal membrane characteristics. Third, we included congestive heart failure in the analysis. We could not analyze the incidence of congestive heart failure separately because it may be underestimated early, leading to a lack of data to evaluate cardiac function in these patients. Fourth, we could not evaluate the true economic burden of FO in patients on PD on health expenditure because the cost per admission of early FO paid by the NHSO, according to the adjusted RW, did not represent the true expenses and did not include the indirect cost. Fifth, the number of patients with persistent and severe FO may be underestimated. We diagnosed severe FO by the cause of hospitalization. In clinical practice, patients with symptoms of FO can be managed at home or at an outpatient clinic during PD visits by performing hypertonic PD solution exchanges. Finally, the population of this study from voluntary data registration in the DPEX leads to selection bias, resulting in the inability to calculate the incidence of FO.

Although there are limitations, our study provides essential information to guide clinical practice and health care policy. The effect of timing and frequency of fluid overload (FO) hospitalizations on mortality are clinical indicators for the needs of intensive fluid management, especially in patients with advanced age, diabetes, and high comorbidities.

Continuous monitoring, early intervention, and tailored management strategies are crucial for the improvement of FO in patients on PD. Health care providers should implement routine assessments, provide patients education, evaluate peritoneal membrane characteristics, adjust appropriate PD prescription, analyze causes of FO, and reduce hospitalizations as part of the quality improvement process in fluid management. Policymakers should consider investing in building health literacy programs for patients, including providing more choices of PD solutions specific to those who need them. Developing new PD solutions with varying sodium levels in the future may be another method for better managing FO in patients with complex conditions.

The timing and frequency of FO development significantly affect patient survival and mortality rates. Patients on PD with comprehensive demographic and clinical profiles need effective and aggressive management to control FO. It is crucial to improve outcomes in patients on PD by implementing processes to achieve fluid balance through early intervention, continuous monitoring, and tailored management strategies.

## Supplementary Material

SUPPLEMENTARY MATERIAL

## Data Availability

Anonymized data created for the study are or will be available in a persistent repository upon publication. Analyzable Data. Send the request to review the data to email: siribha@g.swu.ac.th.

## Supplemental Material

Supplemental Table 1. Distribution of PD centers and number of patients classified according to the region of country.

Supplemental Figure 1. Definition of cumulative fluid overload–free time interval.

Supplemental Table 2. Peritonitis rate.

Supplemental Table 3. Baseline characteristics of patients on PD with a history of admission for fluid overload according to time interval from starting PD to first admission because of fluid overload.
