# Supplementary material for: Effect of Time Interval and Frequency of Hospitalization Because of Fluid Overload on Survival in Peritoneal Dialysis: Thailand Experience
Source: Kidney360. 2024 Sep 11;5(11):1675–82. doi: 10.34067/KID.0000000576 (PMC12282628; doi:10.34067/KID.0000000576)
Supplement: SUPPLEMENTARY MATERIAL [file kidney360-5-1675-s001.pdf]

ASN American Society of Nephrology JASN Journal of the American Society of Nephrology KIDNEY360 Accessing Our World From Every Angle CJASN Clinical Journal of the American Society of Nephrology

ASN Journal Disclosure Form

As per ASN journal policy, I have disclosed any financial relationships or commitments I have held in the past 36 months as included below. I have listed my Current Employer below to indicate there is a relationship requiring disclosure. If no relationship exists, my Current Employer is not listed.

S. Changsirikulchai reports the following:
Employer: Srinakharinwirot University; Honoraria: Baxter, Astra-Zeneca.; Advisory or Leadership Role: Nephrology Society Of Thailand, Finance Committee of ISPD; and Other Interests or Relationships: Faculty of Medicine, Srinakharinwirot University; Nephrology Society of Thailand; Thai Transplantation Society.

I understand that the information above will be published within the journal article, if accepted, and that failure to comply and/or to accurately and completely report the potential financial conflicts of interest could lead to the following: 1) Prior to publication, article rejection, or 2) Post-publication, sanctions ranging from, but not limited to, issuing a correction, reporting the inaccurate information to the authors' institution, banning authors from submitting work to ASN journals for varying lengths of time, and/or retraction of the published work.

Name: Siribha Changsirikulchai
Manuscript ID: K360-2024-000460R1
Manuscript Title: Impact of Time-interval and Frequency of Hospitalization Due to Fluid Overload on Survival in Peritoneal Dialysis: Thailand Experience
Date of Completion: August 12, 2024
Disclosure Updated Date: August 12, 2024

ASN American Society of Nephrology | JASN Journal of the American Society of Nephrology | KIDNEY360 Accessing Our World From Every Angle | CJASN Clinical Journal of the American Society of Nephrology

ASN Journal Disclosure Form

As per ASN journal policy, I have disclosed any financial relationships or commitments I have held in the past 36 months as included below. I have listed my Current Employer below to indicate there is a relationship requiring disclosure. If no relationship exists, my Current Employer is not listed.

P. Sangthawan reports the following:
Employer: Prince of Songkla University Hospital; Honoraria: Baxter; Advisory or Leadership Role: Thai Otsuka; Speakers Bureau: Astra Zeneka, Baxter; and Other Interests or Relationships: Member of thailand kidney society.

I understand that the information above will be published within the journal article, if accepted, and that failure to comply and/or to accurately and completely report the potential financial conflicts of interest could lead to the following: 1) Prior to publication, article rejection, or 2) Post-publication, sanctions ranging from, but not limited to, issuing a correction, reporting the inaccurate information to the authors' institution, banning authors from submitting work to ASN journals for varying lengths of time, and/or retraction of the published work.

Name: Pornpen Sangthawan
Manuscript ID: K360-2024-000460R1
Manuscript Title: Impact of Time-interval and Frequency of Hospitalization Due to Fluid Overload on Survival in Peritoneal Dialysis: Thailand Experience
Date of Completion: August 12, 2024
Disclosure Updated Date: August 12, 2024

ASN American Society of Nephrology
JASN Journal of the American Society of Nephrology
KIDNEY360 Accessing Our World From Every Angle
CJASN Clinical Journal of the American Society of Nephrology

ASN Journal Disclosure Form

As per ASN journal policy, I have disclosed any financial relationships or commitments I have held in the past 36 months as included below. I have listed my Current Employer below to indicate there is a relationship requiring disclosure. If no relationship exists, my Current Employer is not listed.

B. Thinkamrop reports the following:
Employer: Khon Kaen University

I understand that the information above will be published within the journal article, if accepted, and that failure to comply and/or to accurately and completely report the potential financial conflicts of interest could lead to the following: 1) Prior to publication, article rejection, or 2) Post-publication, sanctions ranging from, but not limited to, issuing a correction, reporting the inaccurate information to the authors' institution, banning authors from submitting work to ASN journals for varying lengths of time, and/or retraction of the published work.

Name: Bandit Thinkamrop
Manuscript ID: K360-2024-000460R1
Manuscript Title: Impact of Time-interval and Frequency of Hospitalization Due to Fluid Overload on Survival in Peritoneal Dialysis: Thailand Experience" to Kidney360
Date of Completion: August 13, 2024
Disclosure Updated Date: August 13, 2024

ASN American Society of Nephrology · JASN Journal of the American Society of Nephrology · KIDNEY360 Accessing Our World From Every Angle · CJASN Clinical Journal of the American Society of Nephrology

ASN Journal Disclosure Form

As per ASN journal policy, I have disclosed any financial relationships or commitments I have held in the past 36 months as included below. I have listed my Current Employer below to indicate there is a relationship requiring disclosure. If no relationship exists, my Current Employer is not listed.

J. Thinkhamrop has nothing to disclose.

I understand that the information above will be published within the journal article, if accepted, and that failure to comply and/or to accurately and completely report the potential financial conflicts of interest could lead to the following: 1) Prior to publication, article rejection, or 2) Post-publication, sanctions ranging from, but not limited to, issuing a correction, reporting the inaccurate information to the authors' institution, banning authors from submitting work to ASN journals for varying lengths of time, and/or retraction of the published work.

Name: Jadsada - Thinkhamrop
Manuscript ID: K360-2024-000460R1
Manuscript Title: Impact of Time-interval and Frequency of Hospitalization Due to Fluid Overload on Survival in Peritoneal Dialysis: Thailand Experience
Date of Completion: August 13, 2024
Disclosure Updated Date: August 13, 2024

ASN
American Society of Nephrology
JASN
Journal of the American Society of Nephrology
KIDNEY360
Accessing Our World From Every Angle
CJASN
Clinical Journal of the American Society of Nephrology

ASN Journal Disclosure Form

As per ASN journal policy, I have disclosed any financial relationships or commitments I have held in the past 36 months as included below. I have listed my Current Employer below to indicate there is a relationship requiring disclosure. If no relationship exists, my Current Employer is not listed.

K. Thinkhamrop has nothing to disclose.

I understand that the information above will be published within the journal article, if accepted, and that failure to comply and/or to accurately and completely report the potential financial conflicts of interest could lead to the following: 1) Prior to publication, article rejection, or 2) Post-publication, sanctions ranging from, but not limited to, issuing a correction, reporting the inaccurate information to the authors' institution, banning authors from submitting work to ASN journals for varying lengths of time, and/or retraction of the published work.

Name: Kavin Thinkhamrop
Manuscript ID: K360-2024-000460R1
Manuscript Title: Impact of Time-interval and Frequency of Hospitalization Due to Fluid Overload on Survival in Peritoneal Dialysis: Thailand Experience
Date of Completion: August 13, 2024
Disclosure Updated Date: August 13, 2024

ASN
American Society of Nephrology
JASN
Journal of the American Society of Nephrology
KIDNEY360
Accessing Our World From Every Angle
CJASN
Clinical Journal of the American Society of Nephrology

ASN Journal Disclosure Form

As per ASN journal policy, I have disclosed any financial relationships or commitments I have held in the past 36 months as included below. I have listed my Current Employer below to indicate there is a relationship requiring disclosure. If no relationship exists, my Current Employer is not listed.

J. Thuanman reports the following:
Employer: Khon Kaen University

I understand that the information above will be published within the journal article, if accepted, and that failure to comply and/or to accurately and completely report the potential financial conflicts of interest could lead to the following: 1) Prior to publication, article rejection, or 2) Post-publication, sanctions ranging from, but not limited to, issuing a correction, reporting the inaccurate information to the authors' institution, banning authors from submitting work to ASN journals for varying lengths of time, and/or retraction of the published work.

Name: Jaruwan Thuanman
Manuscript ID: K360-2024-000460R1
Manuscript Title: Impact of Time-interval and Frequency of Hospitalization Due to Fluid Overload on Survival in Peritoneal Dialysis: Thailand Experience
Date of Completion: August 13, 2024
Disclosure Updated Date: August 13, 2024
