# Supplementary material for: Effect of Time Interval and Frequency of Hospitalization Because of Fluid Overload on Survival in Peritoneal Dialysis: Thailand Experience
Source: Kidney360. 2024 Sep 11;5(11):1675–82. doi: 10.34067/KID.0000000576 (PMC12282628; doi:10.34067/KID.0000000576)
Supplement: SUPPLEMENTARY MATERIAL [file kidney360-5-1675-s002.pdf]

Supplemental Table 1 The distribution of PD centers and number of cases classified according to the region of country

Regions	Number of centers	Number of patients
Center	44	12,058
North	19	2,848
Northeast	16	3,800
East	11	2,840
South	2	166

Supplemental Figure 1 Definition of cumulative fluid overload-free time interval

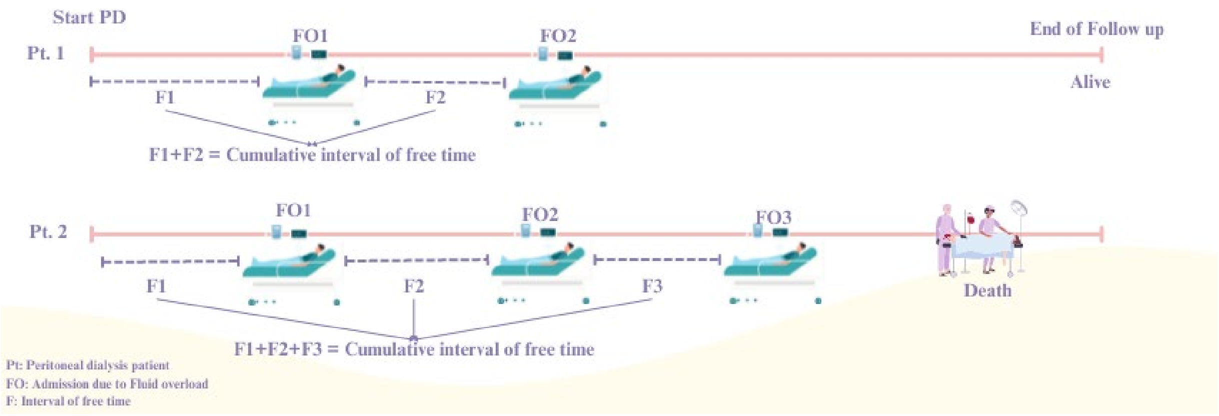

Supplemental Table 2 Peritonitis rate

Follow up year	Number of patients	Person year	peritonitis episodes	peritonitis rate per person year
1	1,858	1,683	537	0.32
2	1,505	1,321	395	0.30
3	1,101	916	289	0.32
4	738	597	175	0.30
5	490	395	156	0.40
6	312	244	67	0.28
7	188	149	30	0.20
8	114	86	18	0.21
9	59	48	11	0.23
10	36	25	2	0.08

Supplemental Table 3 Baseline characteristic of PD patients with a history of admission from fluid overload according to time interval from starting PD to first admission due to fluid overload

**Characteristics (n=1,858)**	**Time interval from starting PD to first admission due to FO**				**p-value**
	**0-12 months (n=1,211)**	**12-24 months (n=256)**	**24-36 months (n=170)**	**More than 36 months (n=221)**	
**Age at PD initiation (IQR)**	56.8 (47.3-64.9)	54.6 (45.7-62.6	56.5 (47.9-64.2)	51.6 (43.8-60.9)	0.054
**Gender n (%)**					0.103
Male	615 (50.8%)	133 (52.0%)	74 (43.5%)	98 (44.3%)	
Female	596 (49.2%)	123 (48.1%)	96 (56.5%)	123 (55.7%)	
**Cause of ESKD n (%)**					
Diabetic nephropathy	670 (55.3%)	147 (57.4%)	86 (50.6%)	91 (41.2%)	0.001
Analgesic nephropathy	131 (10.8%)	16 (6.3%)	9 (5.3%)	12 (5.4%)	0.004
Hypertension	300 (24.8%)	61 (23.8%)	50 (29.4%)	66 (29.9%)	0.232
Obstructive nephropathy	7 (0.6%)	3 (1.2%)	3 (1.8%)	7 (3.2%)	0.005
Others	35 (2.9%)	8 (3.1%)	2 (1.2%)	9 (4.1%)	0.391
Unknown	68 (5.6%)	21 (8.2%)	20 (11.8%)	36 (16.3%)	<0.001
**Comorbidity n (%)**					
Cerebrovascular disease	355 (29.3%)	41 (16.0%)	14 (8.2%)	21 (9.5%)	<0.001
Cardiovascular disease	331 (27.3%)	44 (17.2%)	34 (20.0%)	31 (14.0%)	<0.001
Liver disease	59 (4.9%)	6 (2.3%)	6 (3.5%)	6 (2.7%)	0.197
Gastrointestinal disease	70 (5.8%)	4 (1.6%)	0 (0.0%)	0 (0.0%)	<0.001
Airway disease	72 (6.0%)	9 (3.5%)	9 (5.3%)	15 (6.8%)	0.398
**Number of comorbidity n (%)**					<0.001
1 comorbidity	224 (18.5%)	48 (18.8%)	31 (18.2%)	44 (19.9%)	
2 comorbidities	206 (17.0%)	22 (8.6%)	16 (9.4%)	21 (9.5%)	
3 or more comorbidities	119 (9.8%)	9 (3.5%)	3 (1.8%)	10 (4.5%)	

Abbreviations: PD: Peritoneal dialysis; FO: Fluid overload; ESKD: End-stage of kidney disease; and IQR: Interquartile range

Supplemental Table 4 Baseline characteristic of PD patients with a history of admission from fluid overload according to cumulative fluid overload-free time

**Characteristics** (n=1,035)	**Cumulative fluid overload-free time**				**p-value**
	**0-12 months (n= 460)**	**12-24 months (n= 217)**	**24-36 months (n= 134)**	**More than 36 months (n= 224)**	
**Age at PD initiation (IQR)**	56 (47.3 – 65.4)	54.1 (46.1 – 61.8)	55.5 (47.3 – 62.6)	54.5 (45.3 – 63.5)	0.072
**Gender n (%)**					0.977
Male	233 (50.7%)	110 (50.7%)	65 (48.5%)	113 (50.5%)	
Female	227 (49.3%)	107 (49.3%)	69 (51.5%)	111 (49.5%)	
**Cause of ESKD n (%)**					
Diabetic nephropathy	246 (53.5%)	134 (61.8%)	83 (61.9%)	106 (47.3%)	0.014
Analgesic nephropathy	59 (12.8%)	21 (9.7%)	5 (3.7%)	10 (4.5%)	<0.001
Hypertension	117 (25.5%)	46 (21.2%)	37 (27.6%)	60 (26.8%)	0.547
Obstructive nephropathy	2 (0.4%)	0 (0.0%)	1 (0.8%)	5 (2.2%)	0.003
Others	13 (2.8%)	4 (1.8%)	4 (3.0%)	9 (4.0%)	0.159
Unknown	23 (5.0%)	12 (5.5%)	4 (3.0%)	34 (15.2%)	<0.001
**Comorbidity n (%)**					
Cerebrovascular disease	241 (31.3%)	23 (19.8%)	10 (14.9%)	14 (16.8%)	<0.001
Cardiovascular disease	237 (30.8%)	27 (23.3%)	16 (23.9%)	12 (114.%%)	0.005
Liver disease	45 (5.9%)	2 (1.7%)	1 (1.5%)	3 (3.6%)	0.138
Gastrointestinal disease	44 (5.7%)	2 (1.7%)	0 (0.0%)	0 (0.0%)	0.004
Airway disease	48 (6.2%)	7 (6.0%)	6 (9.0%)	7 (8.4%)	0.650
**Number of comorbidity n (%)**					0.662
1 comorbidity	79 (17.2%)	45 (20.7%)	21 (15.7%)	44 (19.6%)	
2 comorbidities	80 (17.4%)	33 (15.2%)	27 (20.2%)	38 (17.0%)	
3 or more comorbidities	44 (9.6%)	21 (9.7%)	17 (12.7%)	15 (6.7%)	

Abbreviations: PD: Peritoneal dialysis; FO: Fluid overload; ESKD: End-stage of kidney disease; and IQR: Interquartile range
